# Clinical Features Suggestive of Underlying Eosinophilic Granulomatosis With Polyangiitis Before Dupilumab Therapy in Patients With Chronic Rhinosinusitis With Nasal Polyps

**DOI:** 10.7759/cureus.117710

**Published:** 2026-10-09

**Authors:** Ruka Hashimoto, Takumi Kumai, Michihisa Kono, Kenzo Ohara, Miki Takahara

**Affiliations:** 1 Department of Otolaryngology, Head and Neck Surgery, Asahikawa Medical University, Asahikawa, JPN; 2 Medical Oncology, Dana-Farber Cancer Institute, Boston, USA

**Keywords:** chronic rhinosinusitis with nasal polyp, dupilumab, eosinophilic chronic rhinosinusitis, eosinophilic granulomatosis with polyangiitis, peripheral eosinophilia, prodromal egpa

## Abstract

Dupilumab is effective for eosinophilic chronic rhinosinusitis (ECRS); however, eosinophilic granulomatosis with polyangiitis (EGPA) may become clinically apparent after treatment initiation. We retrospectively reviewed three patients with chronic rhinosinusitis with nasal polyps who developed systemic manifestations leading to a diagnosis of EGPA shortly after dupilumab initiation. All three patients had bronchial asthma and peripheral eosinophilia before treatment. EGPA developed within two weeks to two months after dupilumab initiation, with manifestations including fever, arthralgia, peripheral neuropathy, weight loss, and hypertrophic pachymeningitis. Two patients achieved remission with systemic corticosteroids and mepolizumab, whereas one patient with pre-existing myeloperoxidase-antineutrophil cytoplasmic antibody (MPO-ANCA) positivity had a fatal outcome despite intensive immunosuppressive therapy. Their pretreatment courses suggested that systemic eosinophilic disease may already have been evolving before biologic therapy, particularly in the patient with pre-existing MPO-ANCA positivity. Notably, the timing at which EGPA criteria were fulfilled differed between Japan's Ministry of Health, Labour and Welfare (MHLW) diagnostic criteria and the European Alliance of Associations for Rheumatology/American College of Rheumatology (EULAR/ACR) classification criteria in these patients, highlighting that reliance on a single set of criteria may delay recognition of evolving EGPA. These cases emphasize the importance of evaluating unexplained marked eosinophilia, ANCA positivity, and systemic symptoms before initiating dupilumab and of distinguishing transient treatment-associated eosinophilia from emerging systemic vasculitis during follow-up.

## Introduction

Eosinophilic granulomatosis with polyangiitis (EGPA) is a rare systemic necrotizing vasculitis characterized by bronchial asthma, peripheral eosinophilia, and systemic manifestations resulting from eosinophilic inflammation and small-vessel vasculitis [1]. Common clinical manifestations include fever, weight loss, peripheral neuropathy, pulmonary infiltrates, and eosinophilic infiltration of multiple organs.

Recent studies have increasingly recognized the concept of “pre-EGPA,” also referred to as the prodromal phase of EGPA, as the initial stage of EGPA, which is generally divided into the prodromal allergic, eosinophilic, and vasculitic phases [1]. Patients in this phase typically present with eosinophilic airway diseases such as allergic rhinitis, eosinophilic chronic rhinosinusitis (ECRS), and bronchial asthma, accompanied by elevated peripheral eosinophil counts and increased serum IgE levels [1]. Although overt vasculitic manifestations are absent, some patients subsequently progress to systemic vasculitis. Therefore, careful longitudinal monitoring is warranted in patients suspected of being in the prodromal phase of EGPA.

Biologic therapies have dramatically improved the management of type 2 inflammation such as chronic rhinosinusitis with nasal polyps (CRSwNP), many cases of which overlap with ECRS, and severe asthma. Nasal obstruction caused by CRSwNP may contribute to sleep-disordered breathing, including obstructive sleep apnea (OSA), highlighting that CRSwNP can have clinical implications beyond the sinonasal tract and may also affect respiratory function. Dupilumab, an anti-interleukin-4 receptor alpha (IL-4Rα) monoclonal antibody that inhibits IL-4 and IL-13 signaling, has demonstrated remarkable efficacy in reducing nasal polyp burden and improving sinonasal symptoms [2]. Mepolizumab, an anti-IL-5 monoclonal antibody, is another biologic agent that has demonstrated efficacy in both eosinophilic airway diseases and EGPA [3]. Although direct head-to-head comparisons among biologic agents are limited, network meta-analyses suggest that dupilumab may be particularly effective in improving nasal symptoms [4]. Here, we report three patients with ECRS who developed EGPA shortly after dupilumab initiation and discuss clinical features that may facilitate the identification of patients in the prodromal phase of EGPA before the emergence of overt vasculitic manifestations.

## Case presentation

Case 1

A 50-year-old woman presented with recurrent olfactory dysfunction. She had undergone endoscopic sinus surgery for CRSwNP more than 10 years earlier and had a history of bronchial asthma. Myeloperoxidase-antineutrophil cytoplasmic antibody (MPO-ANCA) was negative. Dupilumab therapy was initiated because of recurrent nasal obstruction and nasal polyposis.

Four weeks after dupilumab initiation, the peripheral blood eosinophil percentage increased from 14.8% to 18.1% (reference range, 1-6%), corresponding to an increase in the absolute eosinophil count from 1,110 to 1,170 cells/μL (Figure 1A, Table 1).

**Figure 1:**
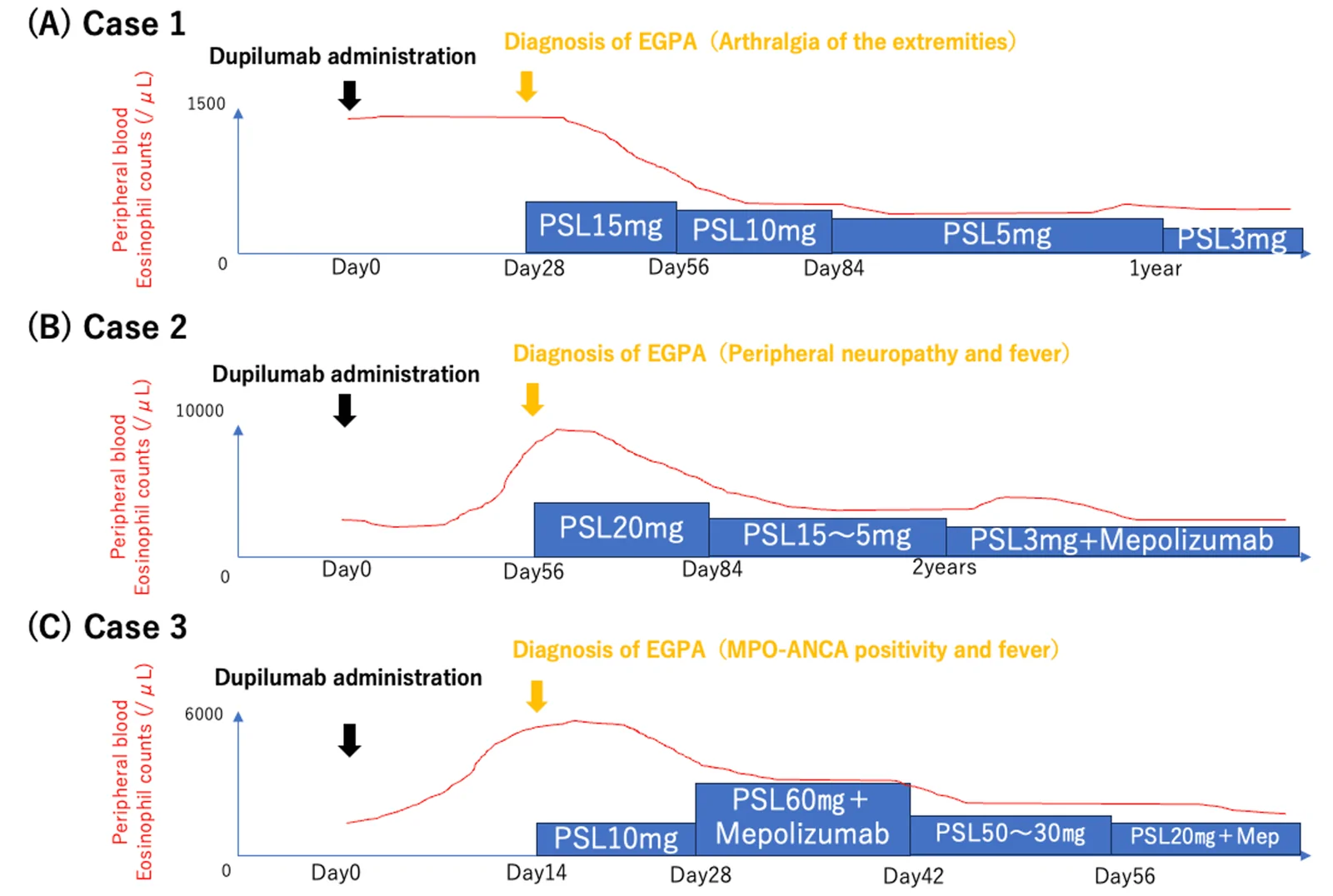
Clinical courses of three patients who developed eosinophilic granulomatosis with polyangiitis after dupilumab therapy. Changes in peripheral blood eosinophil percentages and absolute eosinophil counts after dupilumab administration are shown for Case 1 (A), Case 2 (B), and Case 3 (C). The timing of dupilumab administration, diagnosis of EGPA, and subsequent treatment with prednisolone and/or mepolizumab are indicated. EGPA: eosinophilic granulomatosis with polyangiitis, PSL, prednisolone; Mep, mepolizumab; MPO-ANCA, myeloperoxidase-antineutrophil cytoplasmic antibody.

**Table 1:** Patient characteristics The 2022 EULAR/ACR classification criteria for EGPA assign weighted points to obstructive airway disease (+3), nasal polyps (+3), mononeuritis multiplex (+1), blood eosinophil count ≥1 × 10⁹/L (+5), extravascular eosinophilic-predominant inflammation on biopsy (+2), positive c-ANCA or PR3-ANCA (−3), and hematuria (−1). A cumulative score of ≥6 points is required for classification as EGPA after excluding mimics and establishing a diagnosis of small- or medium-vessel vasculitis [5]. MHLW criteria: The major clinical features include bronchial asthma or allergic rhinitis, peripheral blood eosinophilia, and signs or symptoms of systemic vasculitis. Definite EGPA is diagnosed when all three clinical features are present in the typical clinical course, or when these clinical features are accompanied by characteristic histopathological findings [6]. CRSwNP, chronic rhinosinusitis with nasal polyps; EGPA, eosinophilic granulomatosis with polyangiitis; EULAR/ACR, European Alliance of Associations for Rheumatology/American College of Rheumatology

Feature	Case 1	Case 2	Case 3
Asthma	+	+	+
CRSwNP	+	+	+
Blood eosinophil counts (/μl) before dupilumab	1,110	2,520	1,030
Peak blood eosinophil counts (/μl) after dupilumab	1,170	8,550	4,950
MPO-ANCA before dupilumab	–	–	+
Fever	–	+	+
Weight loss	–	–	+
Arthralgia	+	–	–
Peripheral neuropathy (numbness of the extremities)	–	+	–
Hypertrophic pachymeningitis	–	–	+
Histologic vasculitis	–	–	–
Renal involvement	–	–	–
Pulmonary infiltrate	–	–	–
MHLW EGPA criteria before dupilumab	Not fulfilled	Not fulfilled	Not fulfilled
MHLW EGPA criteria after dupilumab	Fulfilled	Fulfilled	Fulfilled
EULAR/ACR criteria fulfilled (score) before dupilumab	11 points (fulfilled)	11 points (fulfilled)	11 points (fulfilled)
EULAR/ACR criteria fulfilled (score) after dupilumab	11 points (fulfilled)	12 points (fulfilled)	11 points (fulfilled)

She subsequently developed severe polyarthralgia of the extremities that interfered with activities of daily living and was referred to our department for further evaluation. Although histopathological examination did not demonstrate vasculitis, the clinical findings fulfilled the revised Japanese Ministry of Health, Labour and Welfare (MHLW) criteria [5] and 2022 European Alliance of Associations for Rheumatology/American College of Rheumatology classification criteria (EULAR/ACR) [6] for EGPA. Dupilumab was discontinued, and systemic corticosteroid therapy was initiated and managed by the Department of Clinical Immunology. Arthralgia improved within several days, and the patient has remained stable on maintenance prednisolone therapy at 3 mg/day without recurrence.

Case 2

A 51-year-old woman presented with recurrent olfactory dysfunction. She had previously undergone endoscopic sinus surgery for CRSwNP and subsequently developed recurrent nasal obstruction. Her medical history included bronchial asthma and allergic rhinitis. MPO-ANCA was negative. Dupilumab therapy was initiated because of refractory disease.

Two months after dupilumab initiation, the peripheral blood eosinophil percentage increased markedly from 24.5% (2,520 cells/μL) to 55.4% (8,550 cells/μL) (Figure 1B). She subsequently developed recurrent fever and peripheral neuropathy (numbness of the extremities). EGPA was diagnosed clinically by the MHLW and EULAR/ACR criteria; dupilumab was discontinued, and oral prednisolone was initiated at 20 mg/day. Because recurrent fever and numbness of the extremities persisted, suggesting ongoing eosinophilic disease activity, mepolizumab was subsequently introduced. After steroid and anti-IL-5 therapy, nasal polyps markedly regressed, and vasculitic symptoms resolved. The patient remains in remission while receiving low-dose maintenance prednisolone therapy.

Case 3

A 66-year-old man presented with recurrent olfactory dysfunction. He had undergone nasal polypectomy 30 years earlier and bilateral Caldwell-Luc surgery 25 years earlier. Because of persistent olfactory dysfunction, endoscopic sinus surgery had been performed several years before presentation.

Olfactory dysfunction subsequently recurred, and CRSwNP was diagnosed. Concurrent worsening of bronchial asthma prompted initiation of dupilumab therapy by a pulmonologist. Before dupilumab initiation, peripheral blood eosinophils were elevated at 15.3% (1,030 cells/μL), and MPO-ANCA was positive. Two weeks after dupilumab administration, he developed fever and weight loss, and the peripheral blood eosinophil percentage increased further to 22% (4,950 cells/μL, Figure 1C). No hematologic malignancy or other evident secondary cause of eosinophilia was identified clinically. Chest CT demonstrated bronchial wall thickening (Figure 2A).

**Figure 2:**
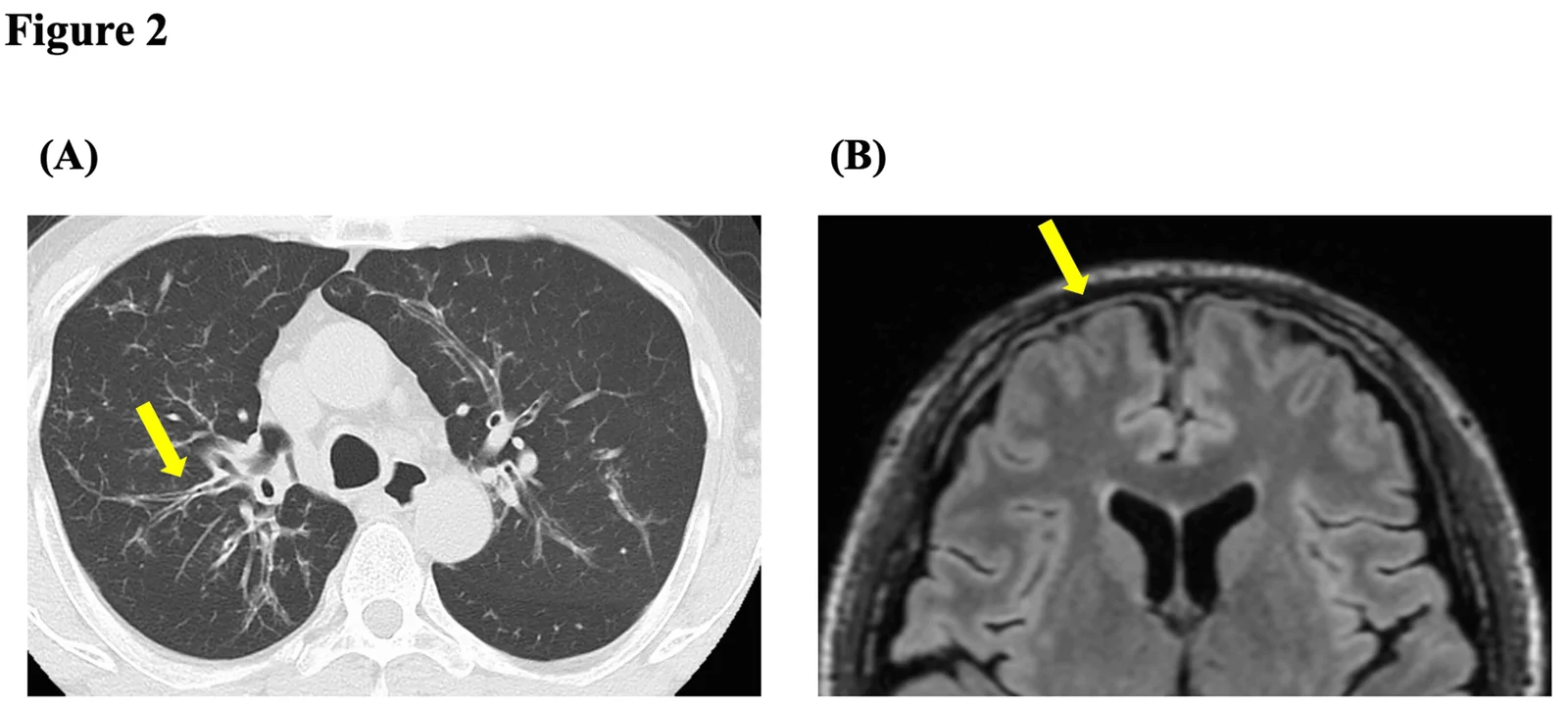
Representative imaging findings in Case 3. (A) Chest computed tomography showing bronchial wall thickening associated with eosinophilic granulomatosis with polyangiitis (arrow). (B) Brain magnetic resonance imaging showing hypertrophic pachymeningitis that developed during the clinical course (arrow).

Based on these findings, EGPA was diagnosed by the MHLW and EULAR/ACR criteria. Dupilumab was discontinued, and treatment with prednisolone at 60 mg/day and mepolizumab was initiated. Bronchial wall thickening improved after treatment. However, headache developed during steroid tapering, and magnetic resonance imaging revealed hypertrophic pachymeningitis (Figure 2B). Despite steroid pulse therapy and intravenous cyclophosphamide, disease control was not achieved. The patient experienced a fatal cardiovascular event 10 months after the diagnosis of EGPA.

Clinical characteristics of patients who developed EGPA after dupilumab therapy

The clinical features of the three patients who developed EGPA after dupilumab therapy are summarized as follows. All patients had bronchial asthma and elevated peripheral blood eosinophil levels before treatment initiation. EGPA developed shortly after dupilumab initiation in all three patients, with an onset ranging from 2 weeks to 2 months after treatment initiation. Clinical manifestations included arthralgia, fever, peripheral neuropathy, numbness of the extremities, weight loss, and other systemic features suggestive of vasculitis. One patient had MPO-ANCA positivity before the emergence of overt vasculitic manifestations and subsequently experienced a severe clinical course complicated by hypertrophic pachymeningitis.

Comparison between patients with and without EGPA after dupilumab therapy

Baseline characteristics were compared between patients who developed EGPA after dupilumab therapy and those who did not, as summarized in Table 2. Peripheral blood eosinophil counts were obtained before initiation of EGPA-specific treatment.

**Table 2:** Baseline characteristics of dupilumab-treated patients with eosinophilic chronic rhinosinusitis according to subsequent development of eosinophilic granulomatosis with polyangiitis. EGPA, eosinophilic granulomatosis with polyangiitis; HPF, high-power field; NSAID, nonsteroidal anti-inflammatory drug.

Parameter	New-onset EGPA (n=3)	Pre-existing EGPA (n=4)	Non-EGPA (n=12)
Sex (male : female)	1:2	2:2	4:8
Age (median)	51 (50-66)	71 (65-76)	50 (31-79)
Asthma	3/3	4/4	9/12
NSAID hypersensitivity	1/3	0/4	6/12
Eosinophilic otitis media	1/3	1/4	1/12
Blood eosinophil counts (/µl, median)	1110 (1030-2520)	1530 (710-4980)	490 (150-1060)
Nasal polyp tissue eosinophils/HPF (median)	200 (70-735)	70 (70-100)	89 (70-500)

No differences were observed in sex distribution, age, prevalence of bronchial asthma, NSAID hypersensitivity, eosinophilic otitis media, or eosinophil infiltration in nasal polyp tissue. However, the patients who subsequently developed EGPA had higher baseline absolute peripheral blood eosinophil counts than those who did not develop EGPA (Figure 3).

**Figure 3:**
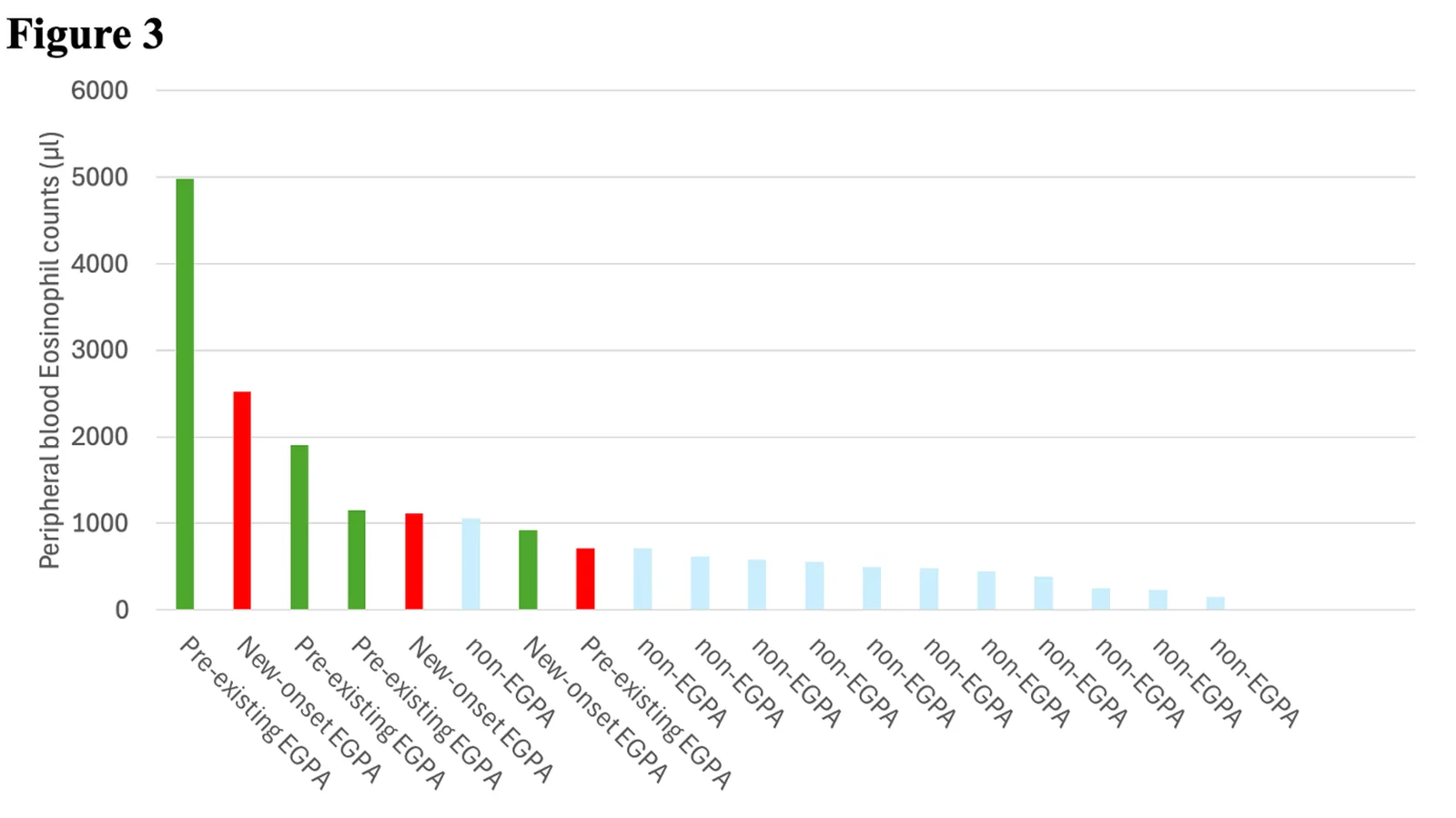
Pretreatment peripheral blood eosinophil counts in patients with pre-existing EGPA, newly diagnosed EGPA after dupilumab initiation, and non-EGPA controls. Peripheral blood eosinophil counts before dupilumab initiation are shown for patients with pre-existing eosinophilic granulomatosis with polyangiitis (EGPA), patients in whom EGPA became clinically apparent after dupilumab initiation, and dupilumab-treated patients who did not develop EGPA during follow-up. Each bar represents an individual patient. EGPA, eosinophilic granulomatosis with polyangiitis.

The three patients in whom EGPA became clinically apparent had relatively high pretreatment absolute eosinophil counts compared with many patients who remained free of EGPA during follow-up. Given the very small number of cases, no inferential statistical analysis or diagnostic threshold was considered appropriate.

## Discussion

In the present study, we encountered three patients with CRSwNP who developed EGPA after initiation of dupilumab therapy. Importantly, all three patients presented in this case series had already fulfilled the ACR/EULAR classification criteria [5] for EGPA before initiation of dupilumab therapy, whereas none fulfilled the MHLW diagnostic criteria [6] at that time. This discrepancy likely reflects the fact that, unlike the ACR/EULAR criteria, the MHLW criteria place greater emphasis on clinical manifestations of vasculitis [5][6]. Accordingly, these cases can be interpreted differently depending on the criteria applied. Based on the MHLW criteria, they may be regarded as cases in which EGPA developed after dupilumab initiation. In contrast, according to the ACR/EULAR criteria, EGPA may already have been present before treatment, with dupilumab therapy temporally associated with the subsequent emergence of overt systemic vasculitic manifestations. In either interpretation, these cases emphasize the importance of reassessing the possibility of EGPA before initiating dupilumab when eosinophilia is accompanied by ANCA positivity or systemic features.

Although dupilumab has shown substantial efficacy in patients with CRSwNP, a small number of EGPA cases have been observed in the phase 3 LIBERTY NP SINUS-52 trial and real-world practice [2,7,8]. Transient peripheral eosinophilia has been reported in approximately 20% of patients receiving dupilumab without requiring therapeutic interventions [7]. In most cases, eosinophil counts gradually decrease over time; however, a subset of patients shows persistent eosinophilia, and some may subsequently develop EGPA. Several mechanisms may underlie this phenomenon. First, discontinuation or tapering of systemic corticosteroids or anti-IL-5 therapy after dupilumab initiation may unmask previously suppressed EGPA. Second, blockade of IL-4/IL-13 signaling may impair eosinophil migration into tissues by suppressing VCAM-1 and eotaxin-3 [7], resulting in increased circulating eosinophils and potentially facilitating the clinical emergence of EGPA. Thus, dupilumab-associated eosinophilia should not always be regarded as a benign transient laboratory finding, particularly in patients with marked baseline eosinophilia or MPO-ANCA positivity, in whom latent EGPA should be carefully considered.

Among currently available biologic agents targeting type 2 inflammatory pathways, dupilumab, which inhibits IL-4/IL-13 signaling, appears to provide particularly favorable efficacy for nasal symptoms in patients with CRSwNP, based on several meta-analyses [4]. In addition, switching from other biologic agents to dupilumab has been reported to improve sinonasal outcomes in selected patients [9]. In line with these findings, EPOS 2020 assigned dupilumab a class 1a recommendation for the treatment of CRSwNP [10].

Given the substantial therapeutic efficacy of dupilumab for CRSwNP, careful recognition of rare but clinically relevant eosinophil-associated complications is essential for optimizing its safe use. Eosinophil-related complications during dupilumab therapy have been reported to occur predominantly within the first six months after treatment initiation [11]. Consistent with this temporal pattern, all three patients in the present series developed EGPA within two months after initiation of dupilumab therapy. Although several cases of EGPA developing after dupilumab therapy have been reported [12], risk factors for progression to definite EGPA remain unclear. In the present study, patients who developed EGPA had elevated peripheral blood absolute eosinophil counts but comparable tissue eosinophils before dupilumab initiation to those who did not develop EGPA. This finding does not support a simple interpretation that dupilumab-induced eosinophilia directly causes EGPA. Instead, marked baseline eosinophilia may reflect an underlying prodromal phase of EGPA in a subset of patients and may be useful for identifying individuals who require close monitoring after dupilumab initiation. The selection of biologic therapy is particularly important in patients who may be in the prodromal phase of EGPA. In our current practice, biologic agents targeting IL-5 or TSLP are considered in patients with marked peripheral eosinophilia or suspected systemic eosinophilic disease. However, the present cases occurred before these agents became available for CRSwNP in our clinical setting, when biologic treatment options were more limited.

Because sinonasal manifestations commonly precede systemic vasculitis by several years, a subset of patients with CRSwNP may be in the prodromal phase of EGPA. In patients with marked peripheral blood eosinophilia, close longitudinal follow-up may enable timely therapeutic intervention before irreversible vasculitic organ damage occurs. The present study has several limitations. First, this was a retrospective, single-center case series with a small number of patients, which limits the generalizability of our findings. Second, histopathological confirmation of vasculitis was not obtained in all cases; therefore, EGPA was diagnosed primarily on the basis of clinical criteria. Finally, because this study was observational, a direct causal relationship between dupilumab therapy and the development of EGPA cannot be established.

## Conclusions

EGPA may become clinically apparent during dupilumab therapy in patients with CRSwNP who have underlying or evolving eosinophilic systemic disease. Although a causal relationship cannot be established, marked or persistent eosinophilia, MPO-ANCA positivity, and systemic symptoms should prompt evaluation for EGPA before and during treatment. Careful distinction between transient dupilumab-associated eosinophilia and emerging systemic vasculitis is important.
